# Factors associated with the uptake of clinical breast examination among women of reproductive age in Lesotho: analysis of a national survey

**DOI:** 10.1186/s12885-023-10566-2

**Published:** 2023-02-01

**Authors:** Agani Afaya, Timothy Tienbia Laari, Abdul Aziz Seidu, Richard Adongo Afaya, Silas Selorm Daniels-Donkor, Vida Nyagre Yakong, Bright Opoku Ahinkorah

**Affiliations:** 1 Mo-Im Kim Nursing Research Institute, College of Nursing, Yonsei University, 50-1, Yonsei-ro, Seodaemun-gu, 03722 Seoul, South Korea; 2 Department of Nursing, School of Nursing and Midwifery, University of Health and Allied Sciences, Ho, Ghana; 3 Presbyterian Primary Health Care (PPHC), Bolgatanga, Ghana; 4 Department of Nursing, Faculty of Allied Health Sciences, College of Health Sciences, Kwame Nkrumah University of Science and Technology, Kumasi, Ghana; 5 College of Public Health, Medical and Veterinary Sciences, James Cook University, Douglas, Australia; 6 REMS Consult Ltd, Takoradi, Ghana; 7 Centre for Gender and Advocacy, Takoradi Technical University, P. O. Box 256, Takoradi, Ghana; 8 Department of Midwifery and Women’s Health, School of Nursing and Midwifery, University for Development Studies, Tamale, Ghana; 9 Department of Nursing, School of Health Sciences, University of Dundee, Scotland, United Kingdom; 10 Department of Preventive Health Nursing, School of Nursing and Midwifery, University for Development Studies, Tamale, Ghana; 11 School of Public Health, Faculty of Health, University of Technology Sydney, Sydney, Australia

**Keywords:** Breast cancer, Clinical breast examination, Reproductive women, Lesotho

## Abstract

**Background:**

In low-resource settings with weak health systems, the WHO recommends clinical breast examination (CBE) as the most cost-effective breast screening modality for women. Evidence shows that biennial CBE leads to significant downstaging of breast cancer in all women. Breast cancer is the second most common cancer among women in Lesotho with a weaker healthcare system and a low breast cancer screening rate. This study investigated the prevalence and factors associated with the uptake of CBE among women of reproductive age in Lesotho.

**Methods:**

This study used cross-sectional data from the 2014 Lesotho Demographic and Health Survey. A sample of 6584 reproductive-age women was included in this study. We conducted both descriptive and multivariable logistic regression analyses. The study results were presented in frequencies, percentages, and adjusted odds ratios (aOR) with their corresponding confidence intervals (CIs).

**Results:**

The prevalence of CBE uptake was 9.73% (95% CI: 8.91, 10.61). Women who were covered by health insurance (aOR = 2.31, 95% CI [1.37, 3.88]), those who were pregnant (aOR = 2.34, 95% CI [1.64, 3.35]), those who had one to three children (aOR = 1.81, 95% CI [1.29,2.52]), and women who frequently read newspapers or magazines (aOR = 1.33, 95% CI [1.02,1.72]) were more likely to undergo CBE than their counterparts. Women who were aware of breast cancer (aOR = 2.54, 95% CI [1.63,3.97]), those who have ever had breast self-examination (BSE) within the last 12 months prior to the study (aOR = 5.30, 95% CI [4.35,6.46]), and those who visited the health facility in the last 12 months prior to the study (aOR = 1.57, 95% CI [1.27,1.95]) were also more likely to undergo CBE than their counterparts. Women residing in the Qacha’s-nek region (aOR = 0.42, 95% CI [0.26,0.67]) were less likely to undergo CBE than those in the Botha-bothe region.

**Conclusion:**

The study found a low prevalence of CBE uptake among reproductive-age women in Lesotho. Factors associated with CBE uptake include health insurance coverage, being pregnant, those who had one to three children, exposure to media, breast cancer awareness, ever had BSE, and those who visited a health facility. To increase CBE uptake, these factors should be considered when designing cancer screening interventions and policies in order to help reduce the burden of breast cancer in Lesotho.

## Background

Globally, breast cancer is the most diagnosed cancer with an estimated number of 2.3 million newly diagnosed cases in 2020 [1]. In sub–Saharan Africa (SSA), breast cancer is the number one cancer and the leading cause of cancer mortality among women. The incidence of breast cancer in SSA is expected to double by 2040 due to population ageing and expansion [2]. The breast cancer survival rates in SSA are poor whereas in high-income countries the survival rates are increasing particularly due to timely diagnosis and effective treatment [2]. Estimates in SSA show that the five-year survival rate is near or almost 50%, meaning that in every two women diagnosed with breast cancer, one will die within five years after diagnosis [3]. The poor survival rate is largely attributed to the advanced-stage diagnosis due to scarce diagnostic equipment, and lack of access to health care in SSA [4–6]. Approximately 80% of women who are diagnosed at the late stages appear in health facilities with locally advanced and metastatic disease in SSA [7]. Substantial efforts have been made to reduce the burden of breast cancer in SSA focusing on improving early detection combined with effective treatment in the early stages in order to improve the survival rate [8]. Despite the efforts made, the participation of women in breast cancer screening remains low [9–11].

The most common screening modalities for breast cancer include breast self-examination (BSE), clinical breast examination (CBE), and mammography. Mammography screening has been observed to be the gold standard in the early detection of breast cancer in high-income countries and reduces breast cancer mortalities by 20–35% [12]. The World Health Organization (WHO) recommends population-based mammography screening for women between the ages of 40 to 75 years in well-resourced settings [13]. However, population-based mammography screening programs are not recommended in resource-limited settings because of it not being cost-effective. In low-resource settings with weak health systems, the WHO recommends CBE as the most cost-effective screening modality for women [13]. A prospective cluster randomized controlled trial for over 20 years in Mumbai, India revealed that biennial CBE led to significant downstaging of breast cancer in all women including those younger than 50 and those aged 50 and older [14]. In the overall population, the researchers revealed a non-significant 15% reduction in breast cancer mortality but a 30% significant reduction in mortality in women aged ≥ 50 [14]. The researchers recommended the implementation of a population-based CBE in resource-limited countries where adequate training of screening providers, prudent monitoring, and quality of performance is assured [14].

Currently, the true burden of breast cancer in Lesotho is not known due to the lack of a national cancer registry. There are no formal breast cancer screening programs implemented in Lesotho. For many years there was no formalized form of treatment for women diagnosed with breast cancer, most cases diagnosed in advanced stages were usually referred to and treated in South Africa or India [15]. In July 2022, a cancer patient received the first chemotherapy treatment ever given in Lesotho. This momentous event was as the result of the Bristol Myers Squibb Foundation’s collaboration with government officials and relevant stakeholders to provide cancer treatment to the people of Lesotho. Though there are lack of healthcare professionals within Lesotho, currently CBE is being provided by doctors and trained nurses [16]. The study by Thabane et al., in Lesotho, explored factors associated with breast cancer screening using combined data on CBE and BSE [15]. Currently, BSE is discouraged due to its harmful effects on women. Evidence from two large trials reported no beneficial effect of BSE but rather reported increased number of benign lesions identified, and an increased number of biopsies performed [17–19]. Due to the above evidence, data on BSE were excluded from this study in order to specifically assess the predictors of CBE uptake. Despite the evidence on CBE, the screening rate among women of reproductive-age in SSA [20–23] including Lesotho remains very low. Although breast cancer incidence is prevalent among postmenopausal women, in the last decade, breast cancer has been observed to occur among women of young age and the trend seems to be increasing in SSA [24]. Therefore, it is also important to consider this cadre of women in the screening process to ensure early detection and treatment. To the best of our knowledge, no study has assessed the factors associated with the uptake of CBE among women of reproductive age in Lesotho. Therefore, efforts geared toward improving early detection and diagnosis among women of reproductive age will be worthwhile. Hence, we investigated the prevalence and factors associated with CBE uptake among women of reproductive age in Lesotho.

## Methods

### Data source

The 2014 Lesotho Demographic Health Survey (LDHS) data were used for this study. The LDHS is a nationwide survey that collects data on a wide range of health-related areas that include maternal and child health, maternal and child mortality, domestic violence, maternal and child nutrition, tuberculosis, malaria, and knowledge about transmission of HIV/ADS. The survey also collected data on breast and cervical cancer together with screening services. The LDHS was executed by the Ministry of Health of Lesotho from 22nd September to 7th December 2014. The survey received financial assistance from the World Bank, the World Health Organization (WHO), United States Agency for International Development (USAID), the United Nations Children’s Fund (UNICEF), the United Nations Population Fund (UNFPA), and other agencies and organizations. Technical assistance for the survey was provided by Inner City Fund (ICF) International through the USAID-funded DHS program.

A multistage (stratified two-stage cluster) sampling design was used to sample participants. The first stage involved the selection of 400 Primary Sampling Units (PSU) where 282 were in rural areas and 118 were in urban areas. The second stage involved systematic sampling of households from each PSU/cluster. The survey interviewed about 9402 individuals from the selected households producing a 99% response rate. During the survey, 6818 reproductive-age women and 3133 men were eligible. Finally, the study included 2931 men (response rate 94%) and 6621 reproductive-age women (response rate 97%). This study included a sample of 6,584 reproductive-age women. The inclusion criteria were women who answered the question: *“Have you had a breast cancer clinical exam to detect breast cancer in the last 12 months?”.* Women who did not respond to the question were excluded. Those who responded to the question but had data missing were excluded from the analysis.

## Study variables

### Outcome variable

The outcome variable for this study was CBE uptake among women of reproductive-age. The question was as follows: “*have you had a breast cancer clinical exam to detect breast cancer in the last 12 months?”* the response was dichotomous: ‘Yes’ was coded as ‘1’ while ‘No’ was coded as ‘0’. Yes = have undergone CBE and No = have not undergone CBE.

### Explanatory variables

Eighteen explanatory variables were included in this study. The study variables were chosen based on related literature [9, 25, 26] and their availability within the dataset. The explanatory variables included women’s age, marital status, educational level, religion, health insurance coverage, parity, currently pregnant, frequency of watching television, frequency of reading newspapers or magazines, frequency of listening to the radio, awareness of breast cancer, practiced BSE in the last 12 months, visited health facility last 12 months, sex of household head, wealth index, type of place of residence, distance to health facility and region.

### Statistical analysis

Data analysis was conducted using Stata version 16. We performed descriptive, bivariate, and multivariate logistic regression analyses. The descriptive analysis was performed to describe the study sample. The bivariate analysis was performed by using the chi-square test to assess the associations between CBE and all the explanatory variables. Bivariate analysis was performed for all the variables that were significant in the chi-square test. The significant variables at the bivariate level were moved to the multivariate logistic regression model. The results for the multivariate logistic regression analysis were presented as adjusted odds ratios (aOR) with 95% confidence intervals (CIs) respectively. A multicollinearity diagnostic test was conducted for all the explanatory variables and none of them had a variance inflation factor more than the rule of thumb (min = 1.02, max = 2.62, mean VIF = 1.32). The study sample was weighted (v005/1,000,000), and the survey set (svy) command in Stata was used in the analyses to account for the survey’s complex nature and the generalizability of the findings.

### Ethical consideration

Permission was sought from MEASURE DHS to use the dataset for this study, which is publicly available. The survey protocol was approved by the Lesotho Ministry of Health Research and Ethics Committee and the Institutional Review Board of ICF International. Written or verbal consent was sought before data collection from participants.

## Results

A total of 6,584 reproductive-age women were sampled for this study. Table 1 depicts the sociodemographic characteristics of the study sample and the proportion of women who had ever undergone CBE. Approximately 21.65% of the women were aged 15–19. Most of the women (53.63%) were married while 51.72% had a secondary level of education. A higher proportion of the women were Christians (97.92%). The majority (97.90%) of the women did not have health insurance coverage. A higher proportion (87.10%) of the women were aware of breast cancer while 37.57% of them have ever performed BSE. Most of the women (65.15%) had male family heads. The majority (63.34%) of the women resided in rural areas while the highest proportion of the women (26.93%) were within the richest wealth index. The majority of the women (28.23%) were from the Maseru region of Lesotho.

**Table 1:** Sociodemographic characteristics of reproductive age women (N = 6,584)

Variables	Weighted N	Weighted %	CBE		
			No (%)90.27%	Yes (%) 9.73%	p-value
**Age (years)**					< 0.001
15–19	1,426	21.65	94.44	5.56	
20–24	1,321	20.06	89.18	10.82	
25–29	1,093	16.60	89.07	10.93	
30–34	949	14.41	88.68	11.32	
35–39	741	11.25	89.53	10.47	
40–44	558	8.48	90.50	9.50	
45–49	496	7.55			
**Marital status**					< 0.001
Never married	2,176	33.05	93.65	6.35	
Married	3,531	53.63	88.63	11.37	
Cohabitation	64	0.96	94.34	5.66	
Widowed	457	6.94	90.81	9.19	
Divorced	357	5.42	89.94	10.06	
**Educational status**					< 0.001
No education	67	1.02	93.75	6.25	
Primary	2,533	38.47	91.79	8.21	
Secondary	3,405	51.72	90.57	9.43	
Higher	578	8.79	83.82	16.18	
**Religion**					0.114
Islam	19	0.29	72.73	27.27	
Hindu	49	0.75	90.77	9.23	
Christianity	6,447	97.92	90.65	9.35	
No religion	69	1.04	85.92	14.08	
**Health insurance coverage**					< 0.001
No	6,446	97.90	90.80	9.20	
Yes	138	2.10	76.85	23.15	
**Parity**					< 0.001
Null	2,064	31.35	94.39	5.61	
1–3	3,533	53.66	88.21	11.79	
4 and above	987	14.99	90.90	9.10	
**Pregnant Status**					< 0.001
No or unsure	6,304	95.74	91.00	9.00	
Yes	280	4.26	80.59	19.41	
**Frequency of reading newspaper or magazine**					< 0.001
Not at all	4,159	63.17	91.94	8.06	
Less than once a week	1,364	20.72	89.17	10.83	
At least once a week	1,061	16.11	86.21	13.79	
**Frequency of listening to the radio**					< 0.001
Not at all	1,541	23.41	93.40	6.60	
Less than once a week	1,059	16.08	90.85	9.15	
At least once a week	3,984	60.51	89.08	10.92	
**Frequency of watching television**					0.059
Not at all	3,641	55.30	91.28	8.72	
Less than once a week	1,002	15.21	89.66	10.34	
At least once a week	1,941	29.49	89.47	10.53	
**Ever heard of breast cancer**					< 0.001
No	849	12.90	97.69	2.31	
Yes	5,735	87.10	89.30	10.70	
**BSE in the last 12 months**					< 0.001
No	4,110	62.43	96.25	3.75	
Yes	2,473	37.57	80.45	19.55	
**Visited health facility last 12 months**					< 0.001
No	2,354	35.76	94.50	5.50	
Yes	4,230	64.24	88.26	11.74	
**Sex of household head**					0.856
Male	4,290	65.15	90.86	9.14	
Female	2,294	34.85	90.02	9.98	
**Wealth index**					< 0.001
Poorest	944	14.35	93.66	6.34	
poorer	1,025	15.57	91.77	8.23	
Middle	1,240	18.83	89.56	10.44	
Richer	1,602	24.33	90.47	9.53	
Richest	1,773	26.93	88.27	11.73	
**Type of place of residence**					0.033
Urban	2,414	36.66	89.48	10.52	
Rural	4,170	63.34	91.11	8.89	
**Distance to a health facility**					0.013
Big problem	1,674	25.43	92.05	7.95	
Not a big problem	4,910	74.57	90.03	9.97	
**Region**					< 0.001
Botha-bothe	382	5.80	89.81	10.19	
Leribe	1,054	16.01	89.73	10.27	
Berea	893	13.56	90.13	9.87	
Maseru	1,859	28.23	88.35	11.65	
Mafeteng	575	8.74	89.07	10.93	
Mohale’s hoek	515	7.82	92.57	7.43	
Quthing	314	4.76	90.79	9.21	
Qacha’s-nek	204	3.09	93.72	6.28	
Mokhotlong	347	5.28	91.53	8.47	
Thaba Tseka	442	6.71	91.83	8.17	

*p-values obtained from chi-square test

### Bivariate association between clinical breast examination and explanatory variables

The overall prevalence of CBE among reproductive-age women in Lesotho was 9.73% (95% CI: 8.91, 10.61). In the bivariate analysis, age (years) (p < 0.001), marital status(p < 0.001), educational level (p < 0.001), health insurance coverage (p < 0.001), parity (p < 0.001), pregnant status (p < 0.001), frequency of reading newspaper or magazine (p < 0.001), frequency of listening to radio (p < 0.001), awareness of breast cancer (p < 0.001), Practiced BSE in the last 12 months (p < 0.001), visited health facility last 12 months (p < 0.001), wealth index (p < 0.001), type of place of residence (p = 0.033), distance to health facility (p = 0.013) and region (p < 0.001) were statistically associated with CBE among reproductive-age women (Table 1).

### Factors associated with the uptake of clinical breast examination among reproductive-age women

Table 2 depicts results from the multivariable logistic regression analysis on the determinants of CBE uptake among women in Lesotho. The results showed that women who had health insurance coverage (aOR = 2.31, 95% CI [1.37, 3.88]) were more likely to undergo CBE than their counterparts. Women who were pregnant (aOR = 2.34, 95% CI [1.64, 3.35]) and those who gave birth between 1 and 3 (aOR = 1.81, 95% CI [1.29,2.52]) were more likely to undergo CBE. Women who frequently read newspapers or magazines (aOR = 1.33, 95% CI [1.02,1.72]) were more likely to have CBE than those who did not. Women who were aware of breast cancer (aOR = 2.54, 95% CI [1.63,3.97]) and those who have ever had BSE within the last 12 months prior to the study (aOR = 5.30, 95% CI [4.35,6.46]) were more likely to undergo CBE than their counterparts. Women who visited the health facility in the last 12 months prior to the study (aOR = 1.57, 95% CI [1.27,1.95]) were more likely to undergo CBE than those who never visited the health facility. Women residing in the Qacha’s-nek region (aOR = 0.42, 95% CI [0.26,0.67]) were less likely to undergo CBE than those in the Botha-bothe region.

**Table 2:** Multivariate analysis of the factors associated with the uptake of clinical breast examination among reproductive-age women

Variable	Model II aOR [95% CI]
**Age (years)**	
15–19	1 [1.00,1.00]
20–24	1.05 [0.76,1.47]
25–29	0.84 [0.59,1.23]
30–34	0.89 [0.61,1.33]
35–39	0.77 [0.50,1.19]
40–44	0.73 [0.46,1.17]
45–49	0.76 [0.46,1.26]
**Marital status**	
Never married	1 [1.00,1.00]
Married	1.22 [0.91,1.65]
Cohabitation	0.49 [0.14,1.78]
Widowed	1.08 [0.69,1.69]
Divorced	0.99 [0.63,1.57]
**Educational status**	
No education	1 [1.00,1.00]
Primary	1.13 [0.43,2.96]
Secondary	1.04 [0.40,2.75]
Higher	1.21 [0.44,3.34]
**Health insurance coverage**	
No	1 [1.00,1.00]
Yes	2.31** [1.37, 3.88]
**Parity**	
Null	1 [1.00,1.00]
1–3	1.81*** [1.29,2.52]
4 and above	1.80* [1.14,2.85]
**Pregnant status**	
No	1 [1.00,1.00]
Yes	2.34*** [1.64, 3.35]
**Frequency of listening to the radio**	
Not at all	1 [1.00,1.00]
Less than once a week	1.06 [0.78,1.44]
At least once a week	1.05 [0.82,1.35]
**Frequency of reading newspaper or magazine**	
Not at all	1 [1.00,1.00]
Less than once a week	1.07 [0.86,1.36]
At least once a week	1.33* [1.02,1.72]
**Ever heard of breast cancer**	
No	1 [1.00,1.00]
Yes	2.54*** [1.63,3.97]
**BSE in the last 12 months**	
No	1 [1.00,1.00]
Yes	5.30*** [4.35,6.46]
**Visited health facility in the last 12 months**	
No	1 [1.00,1.00]
Yes	1.57*** [1.27,1.95]
**Wealth index**	
Poorest	1 [1.00,1.00]
poorer	1.24 [0.88,1.75]
Middle	1.36 [0.96,1.94]
Richer	1.19 [0.82,1.74]
Richest	1.24 [0.82,1.87]
**Type of place of residence**	
Urban	1 [1.00,1.00]
Rural	1.043 [0.83,1.31]
**Distance to a health facility**	
Big problem	1 [1.00,1.00]
Not a big problem	1.10 [0.88,1.37]
**Region**	
Botha-bothe	1 [1.00,1.00]
Leribe	0.67* [0.46,0.99]
Berea	0.61* [0.41,0.90]
Maseru	0.745 [0.52,1.07]
Mafeteng	0.72 [0.48,1.07]
Mohale's hoek	0.52** [0.34,0.81]
Quthing	0.857 [0.57,1.30]
Qacha's-nek	0.42*** [0.26,0.67]
Mokhotlong	0.75 [0.49,1.15]
Thaba Tseka	0.71 [0.46,1.10]

Exponentiated coefficients; 95% confidence intervals in brackets; aOR = adjusted odds ratios; BSE = Breast self-examination; CI = Confidence Interval; ^*^p < 0.05, ^**^p < 0.01, ^***^p < 0.001; 1 = Reference category

## Discussion

This study investigated the prevalence and factors associated with the uptake of CBE using data from the 2014 LDHS. Overall, our study identified the prevalence of CBE uptake to be 9.73%. This finding is consistent with previous studies that reported a 9.1% prevalence of CBE in Nigeria[20], 10.1% in Ghana [21], and 0.9% in Tanzania [22]. The low prevalence of CBE uptake in Lesotho might be due to the inadequate medical infrastructure and services, lack of cancer screening programs and treatment facilities coupled with fewer health professionals [25, 27, 28]. With the increasing breast cancer cases with the disproportionately high mortality rate in SSA, there is therefore the need for advocacy programmes on the importance of CBE to aid improve women’s breast screening rate.

The multivariable analysis of the data identified that having health insurance, being pregnant, giving birth to 1 to 3 children, frequently reading newspapers or magazines, being aware of breast cancer, ever had BSE within the last 12 months prior to the study, visiting the health facility in the last 12 months prior to the study, and residing in the Qacha’s-nek region were important determinants of the uptake of CBE among women of reproductive-age.

Our finding is consistent with a recent study that identified health insurance coverage as a determinant of CBE uptake in Kenya [25] and corroborated by a systematic review in Latin America [29]. This could be ascribed to the fact that health insurance coverage offers women the opportunity to access diagnostic and preventive health care services without financial barriers. Owing to financial and logistical constraints, most countries in SSA are not well placed to implement and sustain screening programmes [30] and the high cost associated with screening could hinder regular uptake of CBE [31, 32]. Therefore, if breast cancer screening is covered by a health insurance scheme, women are most likely to utilise the service [33]. We recommend that women of reproductive age in Lesotho should enrol onto a health insurance scheme to eliminate any financial obstacles to CBE and improve CBE prevalence. Currently as there is no national health insurance but only private insurance schemes [34], we recommend the implementation of a national health insurance scheme that covers breast cancer screening services in Lesotho.

In this study, the odds of undergoing CBE were significantly higher among women who were pregnant. Our finding corroborates a study that reported that Indian women who had one or more pregnancies had better participation in breast cancer screening [35]. Being ever pregnant is a positive predictor of participation in breast cancer screening [36]. The positive relationship between pregnancy and CBE uptake might be due to the frequent utilisation of maternal health care services and therefore exposing them to receive CBE [37]. Therefore, we recommend the integration of CBE into routine antenatal care services in healthcare facilities to further expose more reproductive-age women in Lesotho to CBE.

We found that women who had 1 to 3 children were more likely to undergo CBE. This finding aligns with a previous study in Namibia [38]. A plausible explanation for this observation is that women who have previously given birth are most likely to have had earlier contact with maternal and reproductive health providers, and hence more likely to be acquainted with breast cancer screening. Also, women who have children are familiar with utilizing maternal health services and better positioned to make decisions about CBE uptake compared to women with no childbearing experience. To this effect, we recommend that educational programmes on CBE be targeted at among others, women who have children to further increase CBE uptake.

The odds of CBE were higher among women who were exposed to media, especially newspapers. This finding is similar to previous studies [26, 39]that reported a correlation between mass media coverage and utilisation of breast cancer screening services. Mass media exposure generally increases awareness about breast cancer screening services [39], significantly increases the uptake of such screening services [40], and is recommended for low-resource settings [41]. Accordingly, we recommend the dissemination of CBE awareness and utilisation programmes across the conventional media, especially newspapers/magazines including radio and television as most evidence pinpoints these to enhance breast cancer awareness and screening.

Consistent with previous studies [42–44] women who were aware of breast cancer had higher odds of undergoing CBE. This positive correlation between awareness of breast cancer and CBE may relate to the possibility that women with knowledge are inclined to make better choices to undergo CBE. As such, women are able to balance the merits and demerits of undergoing CBE. Ignorance of breast cancer is detrimental to and negatively impacts screening uptake [45]. Therefore, we recommend a multi-sectorial collaboration to increase community awareness of breast cancer and in effect increase the uptake of CBE among women of reproductive age.

This study finding resonates with studies conducted in the Maldives [46] and Kenya [47] where women who have ever had BSE were more likely to undergo CBE. The exposure of women to CBE may be construed as training on BSE [48] and a low rate of performing BSE results in low CBE uptake [49]. Evidence have demonstrated that the uptake of CBE has strongly improved the utilization of other screening methods such as BSE [50]. This unique correlation between the improved performance of BSE following CBE uptake further highlights the significance of CBE particularly in underdeveloped countries like Lesotho. Owing to this association, we are inclined to advocate for screening by CBE for women of reproductive age in Lesotho.

Our findings further indicate that women who visited the health facility in the last 12 months prior to the study were more likely to undergo CBE which is in line with a previous study [15]. One plausible explanation could be that women who visited healthcare facilities within a year were more exposed to healthcare professionals, had increased access to reproductive health and diagnostic interventions including CBE, and were more likely to benefit from breast cancer screening services in the facilities.

In harmony with earlier studies [15, 25, 51, 52], geographical region was consistently reported to influence CBE. The odds of CBE were lower among women residing in the Qacha’s-nek region than those in the Botha-bothe region. In most underdeveloped countries including Lesotho, there are significant regional disparities in the quality of the healthcare system. Data from the World Bank indicate that the Qacha’s-nek region received the least amount of district-level health expenditure and the lowest total expenditure for health facilities as compared to other regions [28]. Hence, a plausible explanation for our finding might be the resultant limited and inequitable access to health resources and facilities, **educational and socioeconomic differences.**

### Implications for clinicians and policymakers

The study found important factors associated with CBE uptake among reproductive age women in Lesotho. The factors identified in this study could be used by clinicians and relevant stakeholders in implementing public health strategies to promote CBE Uptake among women. We found that breast cancer awareness was a key predictor of CBE uptake, therefore policymakers should consider the implementation of both local and national education programs on breast cancer awareness to improve the low prevalence of CBE uptake among women of reproductive age. Also, various media outlets could be used for breast cancer screening educative programmes. These programmes could increase breast cancer awareness among women which could further have a positive influence on CBE uptake. We recommend that clinicians should incorporate CBE for women attending the health facility for antenatal and postnatal care to enhance its uptake as the study found that parity and pregnancy influence the uptake of CBE.

### Limitations of the study

The study had some limitations. The data of this study was limited to reproductive age women (15–49 years) although breast cancer risk includes women older than 49years therefore the findings cannot be generalised to the wider female population beyond women of reproductive age. The design of the study was cross sectional so we could not establish causality. Also, the study is liable to recall bias due to the self-reported nature of the data.

## Conclusion

The study found a low prevalence of CBE uptake among women of reproductive age in Lesotho. Factors associated with CBE uptake include health insurance coverage, being pregnant, those who had one to three children, exposure to media, breast cancer awareness, ever had BSE, and those who visited the health facility. To increase CBE uptake, these factors should be considered when designing cancer screening interventions and policies in order to help reduce the burden of breast cancer in Lesotho. There is also the need for improved treatment infrastructure to enhance the uptake of CBE among women.

## Data Availability

The datasets used for this study is openly available and can be accessed via https://dhsprogram.com/data/.

## Abbreviations

CBE: clinical breast examination
BSE: breast self examination
SSA: sub-Saharan Africa
LDHS: Lesotho Demographic Health Survey
LMICs: low- and middle-income countries
USAID: United States Agency for International Development
UNFPA: United Nations Population Fund
UNICEF: United Nations Children’s Fund
ICF: Inner City Fund
WHO: World Health Organization
PSU: Primary Sample Units
VIF: Variance Inflation Factor
aOR: adjusted odds ratios
cOR: crude odds ratios
CI: Confidence Interval
